# Impact of single and combined rare diseases on adult inpatient outcomes: a retrospective, cross-sectional study of a large inpatient population

**DOI:** 10.1186/s13023-021-01737-0

**Published:** 2021-02-27

**Authors:** Reka Maria Blazsik, Patrick Emanuel Beeler, Karol Tarcak, Marcus Cheetham, Viktor von Wyl, Holger Dressel

**Affiliations:** 1 Division of Occupational and Environmental Medicine, Epidemiology, Biostatistics and Prevention Institute, University of Zurich and University Hospital Zurich, Hirschengraben 84, 8001 Zurich, Switzerland; 2 Department of Internal Medicine, University Hospital Zurich, Zurich, Switzerland; 3 Department of Epidemiology, Epidemiology, Biostatistics and Prevention Institute, University of Zurich, Zurich, Switzerland; 4 Institute for Implementation Science in Health Care, University of Zurich, Zurich, Switzerland

**Keywords:** Mortality, Length of stay, 30-day readmission, Intensive care unit admission, Rare diseases

## Abstract

**Background:**

Little is known about the impact of rare diseases on inpatient outcomes.

**Objective:**

To compare outcomes of inpatients with 0, 1, or > 1 rare disease. A catalogue of 628 ICD-10 coded rare diseases was applied to count rare diseases.

**Design:**

Retrospective, cross-sectional study.

**Subjects:**

165,908 inpatients, Swiss teaching hospital.

**Main measures:**

Primary outcome: in-hospital mortality. Secondary outcomes: length of stay (LOS), intensive care unit (ICU) admissions, ICU LOS, and 30-day readmissions. Associations with single and combined rare diseases were analyzed by multivariable regression.

**Key results:**

Patients with 1 rare disease were at increased risk of in-hospital death (odds ratio [OR]: 1.80; 95% confidence interval [CI]: 1.67, 1.95), combinations of rare diseases showed stronger associations (OR 2.78; 95% CI 2.39, 3.23). Females with 1 rare disease had an OR of 1.69 (95% CI 1.50, 1.91) for in-hospital death, an OR of 2.99 (95% CI 2.36, 3.79) if they had a combination of rare diseases. Males had an OR of 1.85 (95% CI 1.68, 2.04) and 2.61 (95% CI 2.15, 3.16), respectively. Rare diseases were associated with longer LOS (for 1 and > 1 rare diseases: increase by 28 and 49%), ICU admissions (for 1 and > 1: OR 1.64 [95% CI 1.57, 1.71] and 2.23 [95% CI 2.01, 2.48]), longer ICU LOS (for 1 and > 1 rare diseases: increase by 14 and 40%), and 30-day readmissions (for 1 and > 1: OR 1.57 [95% CI 1.47, 1.68] and 1.64 [95% CI 1.37, 1.96]).

**Conclusions:**

Rare diseases are independently associated with worse inpatient outcomes. This might be the first study suggesting even stronger associations of combined rare diseases with in-hospital deaths, increased LOS, ICU admissions, increased ICU LOS, and 30-day readmissions.

## Introduction

Rare diseases are a diverse group of diseases with a low prevalence. The defined prevalence thresholds of rare diseases vary across references from 5 to 76 cases/100,000 people [1], that is, rare diseases affect a small fraction of the population. Respective estimates range from 3.5–6.2% [2, 3] for the general population [4, 5].

From an epidemiological and clinical viewpoint, rare diseases share some characteristics and challenges [2] that are fundamentally different from those of more common diseases [6]. Patients with rare diseases are geographically widely dispersed, there is a scarcity of clinical expertise and expert centers [6], the patients frequently face misdiagnosis and diagnostic delays [7], and many rare diseases are incurable to date [8].

The fraction of studies into general health indicators and clinical outcomes (i.e., mortality or health-care utilization) of rare diseases in comparison with more frequent conditions is rather small [9]. Some research groups found a disparity between the few patients with rare diseases and their high combined healthcare costs [10, 11]. In general, however, little is known about the clinical impact of rare diseases among inpatients. To our knowledge, no study investigated the effect of combinations of rare diseases on clinical outcomes in the inpatient setting.

Therefore, we examined the impact of the presence of single rare diseases and combinations of rare diseases on inpatient outcomes, focusing on generalizable clinical end points and healthcare utilization. In this context, we studied associations of rare diseases with (i) in-hospital mortality, (ii) increased length of stay (LOS), (iii) intensive care unit (ICU) admissions, (iv) increased ICU LOS, and (v) 30-day readmissions.

## Methods

### Design and study period

We conducted a retrospective, cross-sectional study of routinely prospectively collected electronic health record data of all patients discharged from a Swiss teaching hospital between August 1^st^, 2009 and August 31^st^, 2017. The present investigation used completely anonymous data and conformed with the local law and the ethical review and research policies. Our study adhered to the STrengthening the Reporting of OBservational studies in Epidemiology (STROBE) guidelines [12].

### Setting

The study was performed at a Swiss tertiary care academic medical center with approximately 850 beds and over 35,000 admissions per year. It covers all clinical specialties except orthopedic surgery and pediatrics. Our dataset was derived from a hospital caring for adult patients, and we therefore measured the impact of rare diseases on adult inpatient outcomes, independent of when in the patient’s life the rare disease originated.

### Participants with their stays and diagnoses

**Fig. 1:**
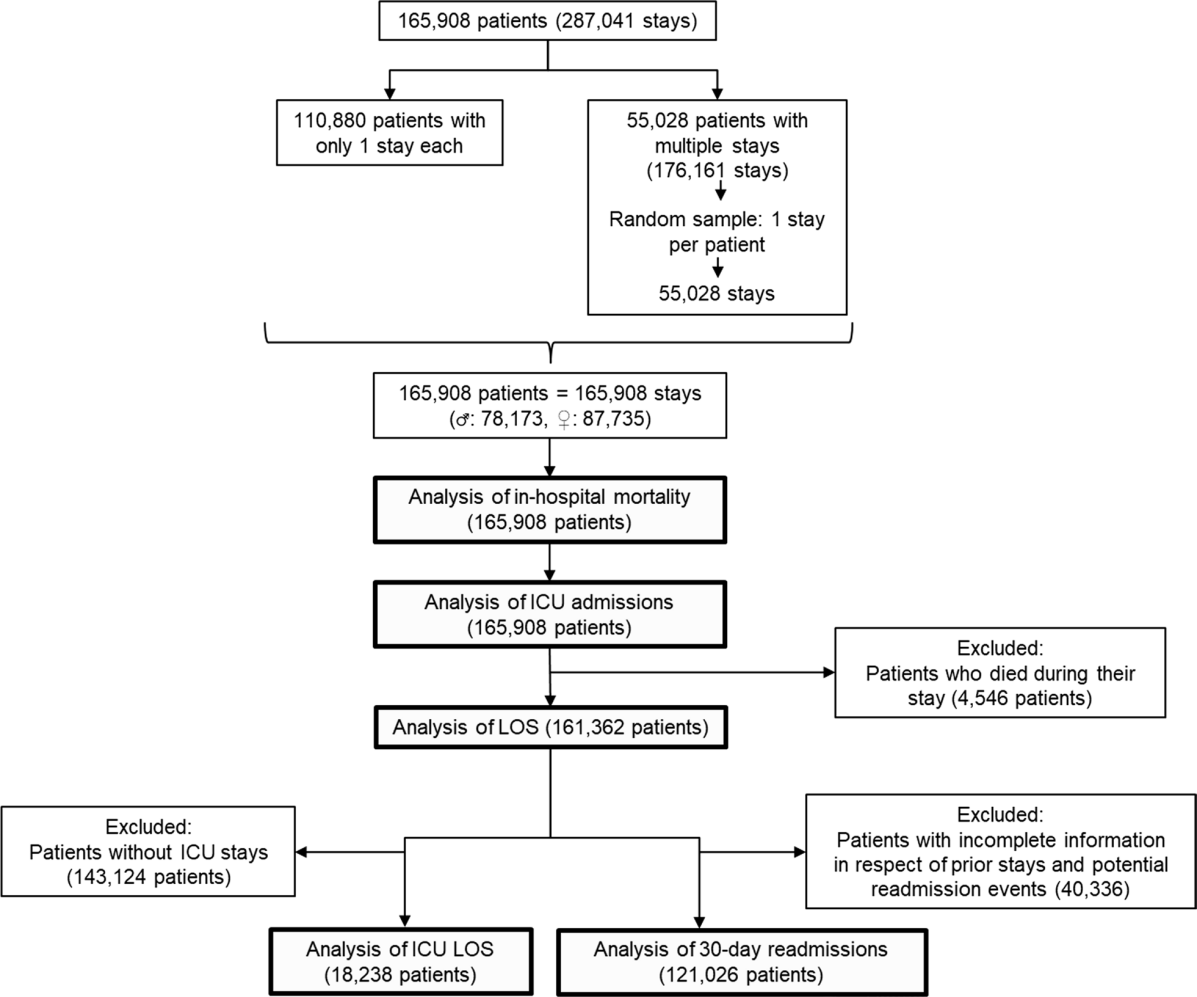
Patient flow diagram and performed outcome analyses

As shown in Fig. 1, we included all adult patients (aged ≥ 18) who had at least one hospital stay during the study period, and only stays with at least one diagnosis were considered.

Two thirds of the patients (110,880patients) stayed once in the institution during the study period, whereas the remaining third (55,028patients) had two or more stays. In respect of the latter group of inpatients who stayed multiple times, the analyzed stays were *randomly* selected to avoid selection bias and to prevent prevalence errors due to patients with rare diseases who stayed multiple times. Thereby we ended up analyzing a total of 165,908 patients with one stay each.

The physicians in charge of the patients assigned and updated all diagnoses over the course of the hospital stay. After discharge of the patients, professional coders assigned ICD-10 codes to each diagnosis (ICD: International Classification of Diseases, WHO, Geneva, Switzerland).

### Main outcomes and measures

The primary outcome was the association of rare diseases with in-hospital mortality. All 165,908 patient stays were considered in this analysis.

We analyzed four secondary outcomes. The first one addressed was the association of rare diseases with ICU admissions. For all remaining secondary outcomes, stays during which the patient died were excluded. Additional secondary outcomes analyzed were LOS (i.e. LOS in the hospital if not otherwise specified), the LOS in the ICU, and readmissions within 30 days after patient discharge.

For the outcome LOS in the ICU, only stays that featured ICU admissions were considered. For the outcome 30-day readmission, only stays with complete information in respect of the number of previous stays within the past two years before admission as well as complete information on potential readmission events were considered [13].

### Exposure

The exposure of interest was the presence of either a single rare disease or a combination of rare diseases. To identify patients with at least one rare disease and to count the total number or rare diseases per patient, we assembled a broad catalogue of ICD-10 coded rare diseases. The sources, developmental steps and iterative improvements of that catalogue is detailed below.

### Catalogue of ICD-10 coded rare diseases

The Orphanet/Orphadata websites provide a catalogue with more than 10,000 rare diseases, of which 8,015 are mapped to a total of 2,075 distinct ICD-10 codes [14–17]. Of those ICD-10 codes, the most frequently found in our study population was E11.9, supposedly coding for *Maturity Onset Diabetes of the Young* (MODY) according to Orphadata. In fact, however, E11.9 officially codes for *Type 2 diabetes mellitus without complications*, which is not rare. We therefore checked whether Orphadata’s descriptions of their ICD-10 codes were equivalent to the official WHO descriptions of the respective ICD-10 codes.

All confirmed ICD-10 codes were then added to the previously published catalogue by Walker et al. [11]. Thereby, we were able to expand the latter by 160 rare diseases, among them 79 rare infectious diseases that were originally excluded. Further steps to improve the new catalogue were performed. Codes were truncated to three or four digits if possible, e.g. Q05.0 to Q05.9 were covered by Q05 *Spina bifida* and therefore replaced by that single code.

All codes in the new catalogue were considered as “wildcards” that can fit in longer, more specific ICD-10 codes found among our inpatients, but not vice versa: On the one hand, the ICD-10 codes of our study population’s diagnoses were truncated to match the catalogue codes (e.g. if a patient had the code D57.1 *Sickle-cell anaemia without crisis*, that code would be truncated to D57 in order to match the catalogue code D57 *Sickle-cell disorders*, and the patient would thus be considered as having a rare disease). On the other hand, the catalogue codes were never truncated to match the study population’s ICD-10 codes (e.g. if a patient had the code A92 *Other mosquito-borne viral fevers*, the catalogue code A92.4 *Rift Valley fever* would not be truncated to A92, hence the patient’s code would not match and the patient would not be considered as suffering from *Rift Valley fever*).

Finally, with a commonly used threshold for the general population [1], we conservatively double-checked in detail all codes with a prevalence among our inpatients of ≥ 1/2,000, which triggered further fine-tuning, e.g. the codes subsumed under *Cranial neuralgia* in the Walker catalogue (G50 to G53) received their official WHO descriptions instead.

Of a total of 628 distinct ICD-10 coded rare diseases in our new catalogue (Additional file 1: available with this publication as online supplementary material), 437 (70%) were found at least once in the study population. The patients were grouped into three categories based on the number of rare diseases they had (0, 1, > 1).

### Co-variables

All regression models were adjusted for age group, sex, the calendar year of hospital discharge, and diagnosis count of non-rare diseases [18] to control for disease burden. The readmission analyses controlled for the number of previous stays in the past two years and for the LOS, in addition to the co-variables mentioned.

### Statistical analysis

For descriptive analyses, categorical variables are presented as counts and percentages. Continuous variables with non-normal distributions are presented as medians and interquartile ranges. For the ICU LOS, due to the usually very low number of days in the ICU, we additionally present the mean (with standard deviation). Chi-squared tests were used to compare categorical variables, Kruskal–Wallis tests to compare continuous variables between patient groups.

Associations of single or combined rare diseases with clinical outcomes were analyzed by means of multivariable logistic regression models for in-hospital mortality, ICU admissions, and 30-day readmissions, and multivariable linear regression models for LOS, and LOS in the ICU.

We used the natural logarithm to transform the skewed outcome variables LOS and LOS in the ICU, described in more detail elsewhere [13]. The estimated coefficients were back-transformed by exponentiation of the coefficients. The back-transformed values can be interpreted as percentage increases or decreases.

Analyses were performed with R, version 4.0.2 (R Foundation for Statistical Computing, Vienna, Austria).

## Results

A total of 165,908 patients were included in our study (Fig. 1.). Table 1 illustrates the baseline characteristics of the patients stratified by their number of rare diseases. 146,804 patients had no rare diseases, whereas 19,104 (11.5%) had one or a combination of rare diseases.

**Table 1:** The baseline characteristics of the patients stratified by their number of rare diseases

Number of rare diseases per patient	0	1	> 1
Number of patients per group	146,804	17,051	2053
Demographics			
Age groups (%)			
18–34	36,084 (24.6)	2280 (13.4)	291 (14.2)
35–49	32,924 (22.4)	2905 (17.0)	382 (18.6)
50–64	31,883 (21.7)	4679 (27.4)	593 (28.9)
65–79	31,417 (21.4)	5160 (30.3)	615 (30.0)
80 and older	14,496 (9.9)	2027 (11.9)	172 (8.4)
Sex = M (%)	67,557 (46.0)	9437 (55.3)	1179 (57.4)
Diagnosis count, excluding rare diseases (median [IQR])	4.00 [2.00, 7.00]	5.00 [3.00, 9.00]	8.00 [4.00, 13.00]
Rare diseases			
Certain infectious and parasitic diseases (%)	0 (0.0)	516 (3.0)	192 (9.4)
Neoplasms (%)	0 (0.0)	3035 (17.8)	590 (28.7)
Diseases of the blood and blood-forming organs and certain disorders involving the immune mechanism (%)	0 (0.0)	609 (3.6)	159 (7.7)
Endocrine nutritional and metabolic diseases (%)	0 (0.0)	1772 (10.4)	467 (22.7)
Mental and behavioural disorders (%)	0 (0.0)	22 (0.1)	6 (0.3)
Diseases of the nervous system (%)	0 (0.0)	3357 (19.7)	574 (28.0)
Diseases of the eye and adnexa (%)	0 (0.0)	401 (2.4)	49 (2.4)
Diseases of the ear and mastoid process (%)	0 (0.0)	169 (1.0)	30 (1.5)
Diseases of the circulatory system (%)	0 (0.0)	1551 (9.1)	393 (19.1)
Diseases of the respiratory system (%)	0 (0.0)	210 (1.2)	57 (2.8)
Diseases of the digestive system (%)	0 (0.0)	655 (3.8)	234 (11.4)
Diseases of the skin and subcutaneous tissue (%)	0 (0.0)	307 (1.8)	56 (2.7)
Diseases of the musculoskeletal system and connective tissue (%)	0 (0.0)	1659 (9.7)	280 (13.6)
Diseases of the genitourinary system (%)	0 (0.0)	78 (0.5)	4 (0.2)
Pregnancy childbirth and the puerperium (%)	0 (0.0)	167 (1.0)	6 (0.3)
Congenital malformations, deformations and chromosomal abnormalities (%)	0 (0.0)	1958 (11.5)	460 (22.4)
Symptoms, signs and abnormal clinical and laboratory findings, not elsewhere classified (%)	0 (0.0)	585 (3.4)	162 (7.9)
Outcomes			
Number of patients who died in hospital (%)	3202 (2.2)	1082 (6.3)	262 (12.8)
30-day readmission (%)			
No	137,918 (93.9)	15,364 (90.1)	1819 (88.6)
Unknown	1768 (1.2)	215 (1.3)	29 (1.4)
Yes	7118 (4.8)	1472 (8.6)	205 (10.0)
Length of stay in days (median [IQR])	5.00 [3.00, 8.00]	7.00 [4.00, 14.00]	11.00 [5.00, 20.00]
ICU admissions (%)	16,178 (11.0)	3987 (23.4)	763 (37.2)
Total time spent in ICUs in days (median [IQR])	0.00 [0.00, 0.00]	0.00 [0.00, 0.00]	0.00 [0.00, 2.00]
Total time spent in ICUs in days (mean (SD))	0.44 (2.70)	1.45 (5.63)	3.59 (9.39)

All p-values < 0.001

### Primary end point

The unadjusted logistic regression model indicated an increased in-hospital mortality associated with the presence of a single rare disease (odds ratio [OR] 3.04; 95% confidence interval [CI]: 2.83 to 3.26), and with the presence of combined rare diseases (OR 6.56; 95% CI 5.74 to 7.50). After adjusting for co-variables, the multivariable logistic regression model showed that rare diseases were independently associated with in-hospital mortality (Table 2). Of note, patients with combinations of rare diseases showed substantially stronger associations with in-hospital death: Compared to patients without any rare diseases, patients with combined rare diseases had an OR of 2.78 (95% CI 2.39 to 3.23) for in-hospital death. Adjusted models for a female subgroup resulted in an increased OR of 1.69 (95% CI 1.50 to 1.91) for in-hospital death if they had a single rare disease, and an OR of 2.99 (95% CI 2.36 to 3.79) if they had a combination of rare diseases (not shown). Adjusted models for a male subgroup resulted in OR of 1.85 (95% CI 1.68 to 2.04) and 2.61 (95% CI 2.15 to 3.16), respectively (not shown).

**Table 2:** Multivariable regression models for in-hospital mortality, ICU admissions, LOS, and ICU LOS

Variable	In-hospital death		ICU admission		LOS		ICU LOS	
	OR	(95% CI)	OR	(95% CI)	Exp(B)	(95% CI)	Exp(B)	(95% CI)
Patients without rare diseases	Ref		Ref		Ref		Ref	
Patients with one rare disease	1.80	(1.67,1.95)	1.64	(1.57,1.71)	1.28	(1.27,1.29)	1.14	(1.10,1.18)
Patients with more than one rare disease	2.78	(2.39,3.23)	2.23	(2.01,2.48)	1.49	(1.45,1.54)	1.40	(1.30,1.51)
Patients aged 18–34	Ref		Ref		Ref		Ref	
Patients aged 35–49	2.05	(1.72,2.45)	1.49	(1.40,1.58)	1.07	(1.06,1.08)	1.03	(0.98,1.09)
Patients aged 50–64	3.76	(3.20,4.41)	2.18	(2.06,2.30)	1.10	(1.09,1.11)	0.97	(0.93,1.01)
Patients aged 65–79	4.46	(3.81,5.23)	2.03	(1.92,2.14)	1.04	(1.03,1.05)	0.85	(0.82,0.89)
Patients aged 80 and older	7.88	(6.72,9.25)	1.16	(1.09,1.25)	0.97	(0.95,0.98)	0.71	(0.67,0.75)
Female sex	Ref		Ref		Ref		Ref	
Male sex	1.19	(1.11,1.26)	1.71	(1.66,1.77)	0.95	(0.94,0.95)	0.97	(0.94,0.99)
Number of diagnoses (excluding rare diseases),								
per additional diagnosis	1.20	(1.19,1.20)	1.20	(1.20,1.21)	1.10	(1.10,1.10)	1.12	(1.12,1.12)
Year discharged,								
per additional year	0.91	(0.90,0.92)	0.94	(0.93,0.94)	0.99	(0.99,0.99)	0.95	(0.94,0.95)

ICU: intensive care unit, LOS: length of stay

We ran three sensitivity analyses, (i) to check whether our results would substantially differ when we adjusted for the number of stays each patient had during the study period. This resulted in OR of 1.86 (95% CI 1.72 to 2.01) and 2.98 (95% CI 2.55 to 3.47) for 1 and > 1 rare disease, respectively (not shown). (ii) To assess the influence of concomitant non-rare conditions, we included an interaction term “number of rare diseases” * “number of non-rare diseases”. This resulted in OR of 2.10 (95% CI 1.80 to 2.46) and 4.30 (95% CI 3.05 to 6.07), respectively (not shown). And (iii) to investigate whether the specialty of the clinical unit where patients received treatment confounded our findings, we included an interaction term “number of rare diseases” * “group of clinical units” (i.e. “internal medicine and related units” vs. “surgical units” vs. “other units”). This resulted in OR of 1.77 (95% CI 1.46 to 2.14) and 2.74 (95% CI 1.80 to 4.18), respectively (not shown).

### Secondary end points

Rare diseases were statistically significantly associated with ICU admissions, longer LOS, longer ICU LOS (Table 2) and 30-day readmissions (Table 3). The associations with ICU admissions, longer LOS, and longer ICU LOS were substantially stronger among patients with combinations of rare diseases.

**Table 3:** Multivariable regression model for 30-day readmissions

Variable	30-day readmission	
	OR	(95% CI)
Patients without rare diseases	ref	
Patients with one rare disease	1.57	(1.47,1.68)
Patients with more than one rare disease	1.64	(1.37,1.96)
Patients aged 18–34	ref	
Patients aged 35–49	1.11	(1.02,1.21)
Patients aged 50–64	1.33	(1.23,1.44)
Patients aged 65–79	1.40	(1.29,1.52)
Patients aged 80 and older	1.31	(1.18,1.44)
Female sex	ref	
Male sex	1.26	(1.20,1.33)
Number of diagnoses (excluding rare diseases),		
per additional diagnosis	1.02	(1.02,1.03)
Year discharged,		
per additional year	0.99	(0.98,1.01)
No previous stays (within prior two years)	ref	
1 previous stay (within prior two years)	1.87	(1.74,2.00)
2 previous stays (within prior two years)	2.80	(2.53,3.11)
3 or more previous stays (within prior two years)	5.86	(5.33,6.44)
Length of stay,		
per additional day	1.01	(1.01,1.01)

## Discussion

This study suggests that the presence of rare diseases is independently associated with worse inpatient outcomes, that is, in-hospital mortality, ICU admissions, LOS, ICU LOS, and 30-day readmissions. These findings persist after controlling for the influence of several potentially confounding factors, including demographics and burden of disease [18]. While we observed those associations in patients with a single rare disease, our study might be the first suggesting substantially stronger associations of combined rare diseases with worsening of some inpatient outcomes, especially in-hospital mortality and ICU LOS.

Rare diseases have been found to account for a greater average length of stay than what the general inpatient population shows [10, 11]. Specific rare diseases, like muscular dystrophies, spina bifida and fragile X syndrome were associated with a higher 30-day all-cause readmission rate [19]. Rare diseases seem to be associated with significant economic burden [20] and a disparity was found regarding healthcare costs and the proportion of the population with rare diseases [10, 11].

To our knowledge, this is the first study on inpatients with rare diseases that analyzes associations with five important clinical outcomes. Moreover, we paid special attention to patient groups with more than one rare disease, thereby investigating the possibility of dose–response relationships between the number of rare diseases and clinical outcomes. The only outcome not necessarily suggesting such a dose–response relationship was 30-day readmissions. Whether a rare disease is present seems to influence clinical outcomes of the inpatients and in turn hospital resource utilization, which is of significance for public health and the healthcare system as a whole.

On the one hand, we considered a broad catalogue of ICD-10 coded rare diseases. On the other hand, our proportion of inpatients with rare diseases might be higher than it would be in other Swiss institutions, because our cohort was derived from a tertiary care academic medical center with highly specialized clinical units and experts providing highly specialized care. Many patients are referred to our institution due to the level of specialization also in respect of diagnostic competencies.

The limitations of our study should be taken into account in interpreting our results. A single center study like ours provides a lower generalizability of the results and conclusions than multi-center studies. However, we included all patients with at least one diagnosis, that is, a large, comprehensive and medically diverse inpatient cohort, which may have improved generalizability. Further, ICD codes are mainly added to the health records for billing and administrative purposes but not for research [21], and we cannot rule out that some rare diseases that are predominantly treated in outpatient settings might have been missed in our study. Also, rare diseases are underrepresented in healthcare coding systems and only a modest fraction of rare diseases have codes in the ICD-10 coding system [16]. Still, we considered a broader catalogue of ICD-10 coded rare diseases than any other study we are aware of [10, 11]. The ICD-11 coding system will provide a substantially improved representation of rare diseases [16] than the current system. This will make rare diseases more visible in the coding system, and also, it will hopefully support and enhance epidemiological research regarding rare diseases. Finally, some interesting data were not available in our dataset: (i) We only had information on in-hospital deaths, but we did not know whether a patient died after discharge. In this context, we cannot rule out that some patients or their families preferred a different setting for end-of-life care than that provided in this study. And (ii) insurance status of patients was not available in our dataset and we were therefore unable to control for it in our regression analyses. Nevertheless, Switzerland has a highly rated health system with mandatory health insurance and nearly universal access to health care [22, 23].

The findings of this study suggest that — across a medically diverse adult inpatient population — patients with rare diseases differ from those with more common diseases. These differences manifest in form of worse clinical outcomes which also entails economic consequences. We therefore advocate that patients with rare diseases should receive special attention in the inpatient setting in order to obtain the best possible outcomes. Since our dataset included only adult inpatients, future studies could investigate whether our findings can be replicated in datasets derived from children’s hospitals.

In conclusion, we analyzed a large and diverse inpatient cohort, we considered a broad catalogue of ICD-10 coded rare diseases, and we demonstrated that rare diseases are independently associated with worse clinical outcomes among inpatients. This might be the first study suggesting that patients with combinations of rare diseases are at even higher risk, especially for in-hospital death and increased ICU LOS. Our results have important clinical implications as well as implications for healthcare utilization and costs, and it seems critical that future efforts are undertaken to find ways to improve clinical outcomes of inpatients with rare diseases.

## Supplementary information

**Additional file 1**. Catalogue of ICD-10 coded rare diseases.

## Data Availability

Data are available from the authors upon reasonable request and with permission of the University Hospital Zurich.
